# Fumarate hydratase suppresses glioblastoma progression by restricting fumarate-mediated succination of TRIM17

**DOI:** 10.1016/j.jbc.2026.113401

**Published:** 2026-08-07

**Authors:** Qianqian Ju, Yan Hu, Chiheng Gong, Runze Wu, Mujie Shen, Xuejian Wang, Xin Tang, Jianhong Shen, Cheng Sun

**Affiliations:** 1 Jiangsu Key Laboratory of Tissue Engineering and Neuroregeneration, Key Laboratory of Neuroregeneration of Ministry of Education, Co-innovation Center of Neuroregeneration, School of Medicine, Nantong University, Nantong, China; 2 Department of Biostatistics, Center for Global Health, School of Public Health, Nanjing Medical University, Nanjing, China; 3 Department of Neurosurgery, Affiliated Hospital 2 of Nantong University, Nantong, China; 4 Department of Neurosurgery, Affiliated Hospital of Nantong University, Nantong, China

**Keywords:** fumarate hydratase, fumarate, glioblastoma, succination TRIM17, metabolic reprogramming

## Abstract

Emerging evidence indicates that metabolic reprogramming plays a pivotal role in glioblastoma (GBM) progression. Here, we report that the expression of fumarate hydratase (FH) is significantly reduced in GBM patient samples. FH overexpression suppresses the viability, migration, and invasion of GBM cells, whereas FH knockdown or pharmacological inhibition produces the opposite effects. Consistent with FH downregulation, its substrate fumarate accumulates in GBM cells and clinical tumor tissues. As a result, excess fumarate induces succination of tripartite motif containing 17 (TRIM17) at Cys370, thereby reducing TRIM17 protein stability and impairing its E3 ubiquitin ligase activity. Subsequent functional studies identify TRIM17 as a tumor suppressor in GBM. *In vivo* xenograft models further confirm that FH restrains GBM progression by regulating TRIM17 succination. Collectively, these findings establish FH as a negative regulator of GBM and reveal a fumarate-TRIM17 signaling axis that promotes GBM malignancy through post-translational modification of TRIM17.

Glioblastoma (GBM) is the most aggressive and lethal primary brain tumor in adults, characterized by rapid proliferation, extensive infiltration, and resistance to conventional therapies (1, 2, 3, 4). Despite progress in surgical resection, radiotherapy, and chemotherapy, the prognosis of GBM remains poor, with a median survival of approximately 15 months (4). These clinical challenges highlight the urgent need to elucidate the molecular mechanisms driving GBM progression, with the goal of identifying novel therapeutic targets and improving patient outcomes.

Among the emerging hallmarks of cancer, metabolic reprogramming plays a central role in promoting tumor cell survival, progression, and resistance to both immunotherapy and chemotherapy (5, 6, 7, 8, 9, 10). GBM cells exhibit remarkable plasticity in energy metabolism, particularly involving mitochondrial dysfunction and alterations in the tricarboxylic acid (TCA) cycle (11, 12, 13). Disruption of the TCA cycle not only compromises cellular bioenergetics but also leads to the accumulation of oncometabolites. These metabolites exert pleiotropic effects on tumor biology by interfering with signaling pathways, epigenetic landscapes, and protein homeostasis (14, 15, 16).

Fumarate hydratase (FH), a key enzyme in the TCA cycle, catalyzes the reversible hydration of fumarate to malate within mitochondria (17). Germline mutations in *FH* are known to driver hereditary leiomyomatosis and renal cell carcinoma, where FH loss results in pathological fumarate accumulation and subsequent activation of oncogenic signaling pathways (18, 19). Notably, fumarate acts as an endogenous electrophile that modifies cysteine residues *via* Michael addition, forming stable S-(2-succino)-cysteine (2SC) adducts in a process termed protein succination (20). This post-translational modification disrupts protein structure and function, and has been shown to impair key regulators of redox homeostasis, transcription, and proteostasis (21, 22, 23). Up to data, the role of FH-mediated succination in GBM remains poorly understood.

In the present study, we investigate the clinical significance and biological function of FH in GBM, and explore the molecular link between fumarate accumulation and protein succination in GBM progression. We identify TRIM17, an E3 ubiquitin ligase, as a key succination target of fumarate, and elucidate the effect of FH deficiency-induced TRIM17 succination on its protein stability and biological activity, thus uncovering a novel metabolic regulatory axis in GBM.

## Results

### Low FH expression in glioma predicts poor prognosis and compromises chemotherapeutic outcomes

**Figure 1:**
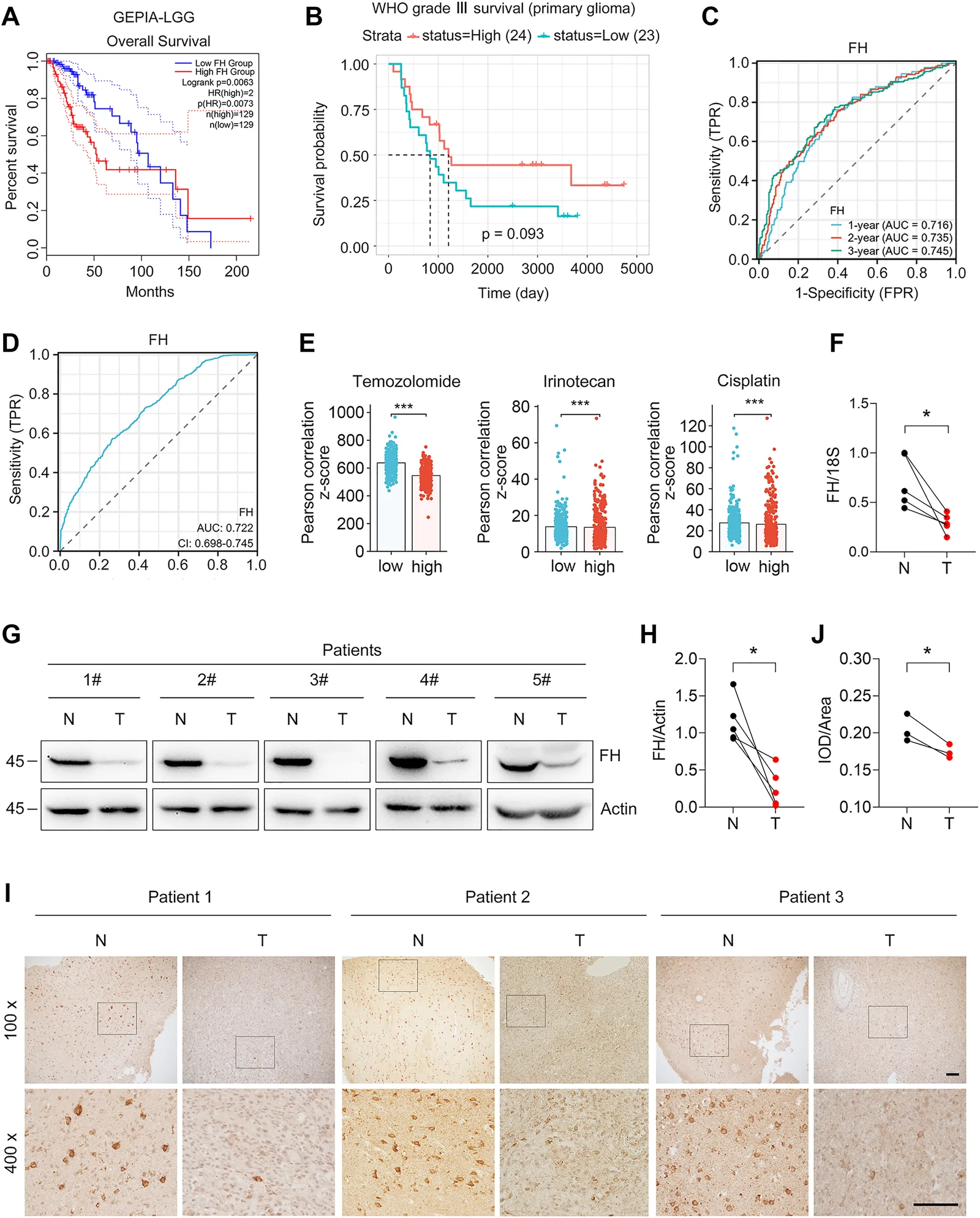
**Low FH expression in GBM is associated with poor prognosis and reduced chemotherapy sensitivity.***A*, Kaplan-Meier survival analysis from the GEPIA database showing that high FH expression correlates with longer OS in low-grade glioma patients. *n=129*. *B*, survival analysis from the CGGA database reveals a trend toward improved OS in primary glioma patients with high FH expression (Log-rank test, *p* = 0.093; *n* = 24 (*high*)/23 (*low*); cutoff: median FH expression). *C*, time-dependent ROC curves evaluating the prognostic value of FH expression for 1-year (*blue*), 2-years (*red*), and 3-years (*green*) OS in glioma patients from the The Cancer Genome Atlas cohort. TPR: true positive rate; FPR: false positive rate. *D*, ROC curve assessing the diagnostic performance of FH expression in distinguishing glioma from normal brain tissues (AUC = 0.722; 95% CI: 0.698–0.745). *E*, chemotherapy sensitivity analysis for temozolomide, irinotecan and cisplatin in FH high- and low-expression glioma patients (The Cancer Genome Atlas cohort; *n* = 351 high/350 *low*). *F*, FH mRNA levels decreased in glioma tissues with comparison to paired adjacent tissues. Gene expression was analyzed by quantitative real time-PCR, and *18S rRNA* was used as an internal control. *G*, Western blot showing decreased FH protein levels in glioma tissues compared to paired adjacent tissues. Actin served as a loading control. T: tumor; *A*: adjacent. *H*, quantification of FH protein expression from (*G*). *n* = 5 biologically independent replicates. *I*, immunohistochemical staining of FH in paired normal and glioma tissues. This scale bar represents 100 μm. *J*, quantitation of FH immunostaining in (*I*) by integral optical density. *n* = 3 biologically independent replicates. Data are presented as mean ± SD. Statistical significance was determined by log-rank test (*A* and *B*), unpaired Student’s *t* test (*E*), and paired Student’s *t* test (*F*, *H* and *J*). ROC analyses in (*C* and *D*) were conducted using the “timeROC” and “pROC” R packages. *∗p* < 0.05. FH, fumarate hydratase; ROC, receiver operating characteristic; OS, overall survival.

To investigate the clinical significance of FH in glioma, we first analyzed publicly available datasets. Kaplan-Meier analysis of the GEPIA low-grade glioma cohort revealed that higher FH expression was significantly associated with prolonged overall survival (OS) (Fig. 1*A*). This finding was further supported by the CGGA dataset, where elevated FH expression correlated with a trend toward improved OS (Fig. 1*B*). Time-dependent receiver operating characteristic (ROC) analysis using the The Cancer Genome Atlas dataset demonstrated that FH expression had moderate prognostic value for 1-, 2-, and 3-years OS (Fig. 1*C*). Additionally, ROC analysis evaluating diagnostic performance indicated that FH expression moderately discriminated glioma from normal brain tissues, with an under the curve of 0.722 (Fig. 1*D*). Moreover, chemotherapy sensitivity analysis further indicated that glioma patients with low FH expression were less responsive to temozolomide, irinotecan, and cisplatin compared to those who with high FH levels (Fig. 1*E*). Consistently, the mRNA and protein levels of FH was markedly reduced in glioma tissues relative to paired adjacent normal tissues (Fig. 1, *F–H*), which was further validated by immunohistochemical staining (Fig. 1, *I* and *J*). Collectively, these results indicate that FH expression is downregulated in glioma and may serve as a potential biomarker of poor prognosis and chemoresistance.

### FH suppresses GBM cell viability and metastasis

**Figure 2:**
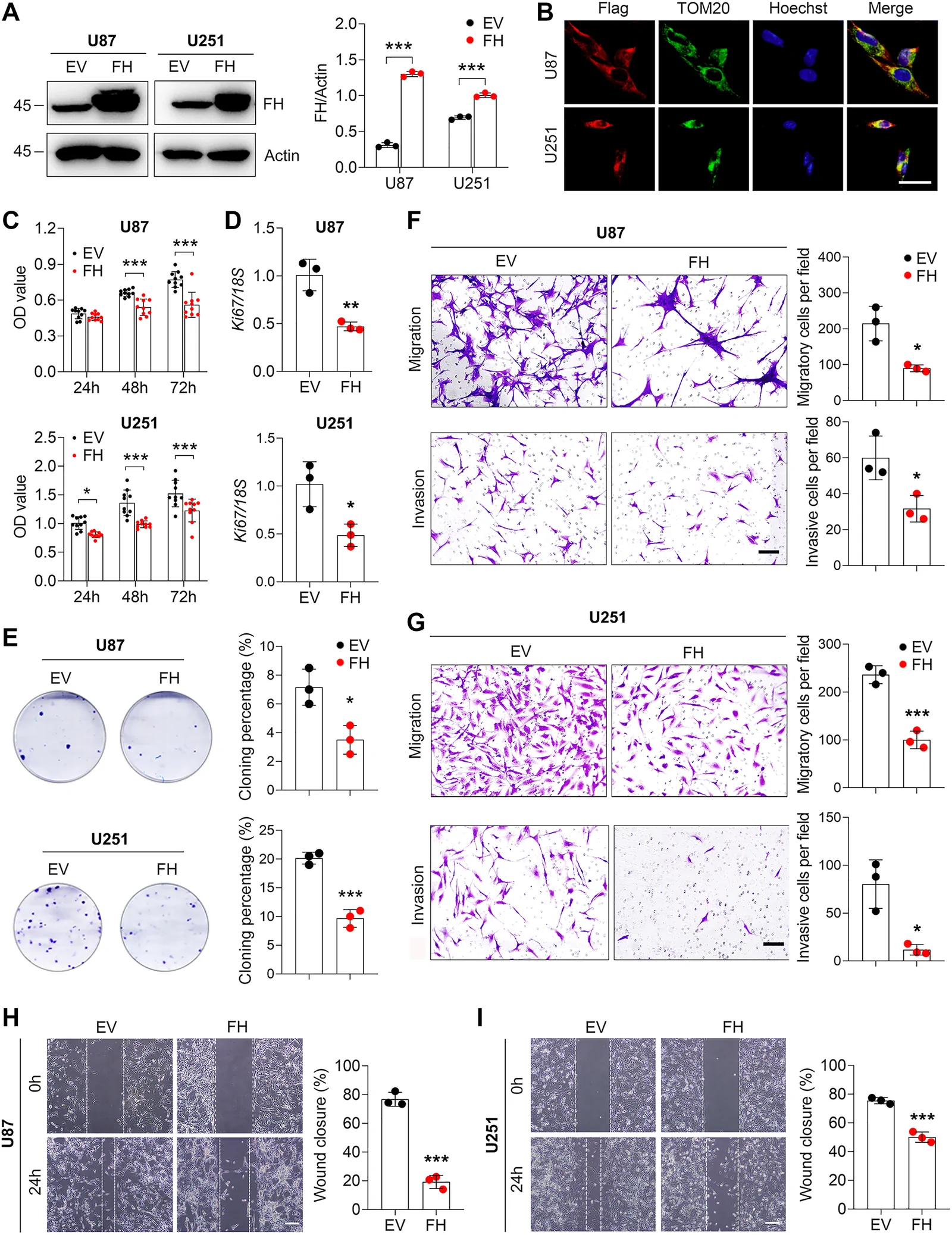
**FH negatively regulates GBM cell growth and metastasis.***A*, FH overexpression in GBM cells. U87 and U251 cells were transfected with a plasmid expressing flag-tagged *FH* for 24 h *n* = *3* biologically independent replicates. *B*, immunofluorescence analysis showing mitochondrial localization of exogenous FH. Cells were stained with anti-Flag and anti-TOM20 antibodies; nuclei were stained with Hoechst. This scale bar reprents20 μm. *C*, FH overexpression reduces GBM cell viability. *n* = *10* biologically independent replicates. *D, Ki67* expression is decreased upon FH overexpression in GBM cells. *n* = *3* biologically independent replicates. *E*, FH overexpression inhibits clonogenic growth in GBM cells. *n* = *3* biologically independent replicates. *F* and *G*, FH overexpression suppresses cell migration and invasion in U87 (*F*) and U251 (*G*) cells, as shown by transwell assays. This scale bar reprents 50 μm. *n* = *3* biologically independent replicates. *H* and *I*, wound healing assay indicates that FH overexpression attenuates cell migration in U87 (*H*) and U251 (*I*) cells. The scale bar represents100 μm. *n* = *3* biologically independent replicates. Protein levels were analyzed by Western blot using Actin as a loading control. Gene expression was analyzed by quantitative real time-PCR with *18S rRNA* as the internal control. Data are presented as mean ± SD. Statistical significance was determined using an unpaired Student’s *t* test. ∗*p* < 0.05, ∗∗*p* < 0.01 and ∗∗∗*p* < 0.001. FH, fumarate hydratase.

Our earlier data indicated that reduced FH expression is closely associated with GBM malignancy, suggesting a potential role for FH in regulating GBM progression. To test this, we overexpressed FH in multiple GBM cell lines, including U87, U251, U118, and GL261 cells (Figs. 2*A* and S1*A*). Immunofluorescence analysis showed that exogenous FH predominantly localized to mitochondria, as evidenced by colocalization with the mitochondrial marker TOM20 (Figs. 2*B* and S1*B*). Functionally, FH overexpression reduced GBM cell viability, proliferation, and clonogenic growth (Figs. 2, *C*–*E* and S1, *C–E*). Transwell assays revealed that FH suppressed both migration and invasion of GBM cells (Figs. 2, *F*, *G* and S1, *F*, *G*), a finding further supported by wound healing assays (Figs. 2, *H*, *I* and S1, *H*, *I*). To verify whether these inhibitory effects depend on the enzymatic activity of FH, we generated two catalytically inactive FH mutants, FH D238H and FH E378K (24), and evaluated their functions in GBM cells. Our data showed that, in contrast to WT FH, both FH D238H and FH E378K failed to inhibit cell viability, migration, and invasion in U251 cells (Fig. S2, *A*–*G*). These results indicate that the suppressive effects of FH on GBM cell viability, migration, and invasion are dependent on its enzymatic activity.

To further validate the tumor-suppressive role of FH, we silenced FH expression in GBM cells using siRNAs. Both quantitative real time-PCR (qRT-PCR) and Western blot analyses confirmed efficient FH knockdown by *FH*-siRNA-3# (Figs. S3, *A*, *B* and S4, *A*, *B*). In contrast to FH overexpression, FH knockdown enhanced GBM cell viability, proliferation, and clonogenic growth (Figs. S3, *C–E* and S4, *C–E*) and promoted cell migration and invasion (Figs. S3, *F*, *G* and S4, *F*, *G*). To corroborate these results, we pharmacologically inhibited FH using the small molecule fumarate hydratase-IN-1. Similar to genetic knockdown, FH inhibition markedly increased GBM cell viability and clonogenic growth (Fig. S5, *A* and *B*) and enhanced migration and invasion (Fig. S6, *A* and *B*). To further validate the observed effects of FH, we performed rescue experiments by re-expressing FH in FH knockdown cells. Our data showed that FH overexpression abolished FH knockdown-induced increases in cell viability, migration, and invasion (Fig. S7, *A*–*G*). Collectively, these findings further support the tumor-suppressive function of FH and suggest that its loss promotes GBM cell malignancy.

### FH suppresses GBM by preventing fumarate-mediated succination and inactivation of TRIM17

**Figure 3:**
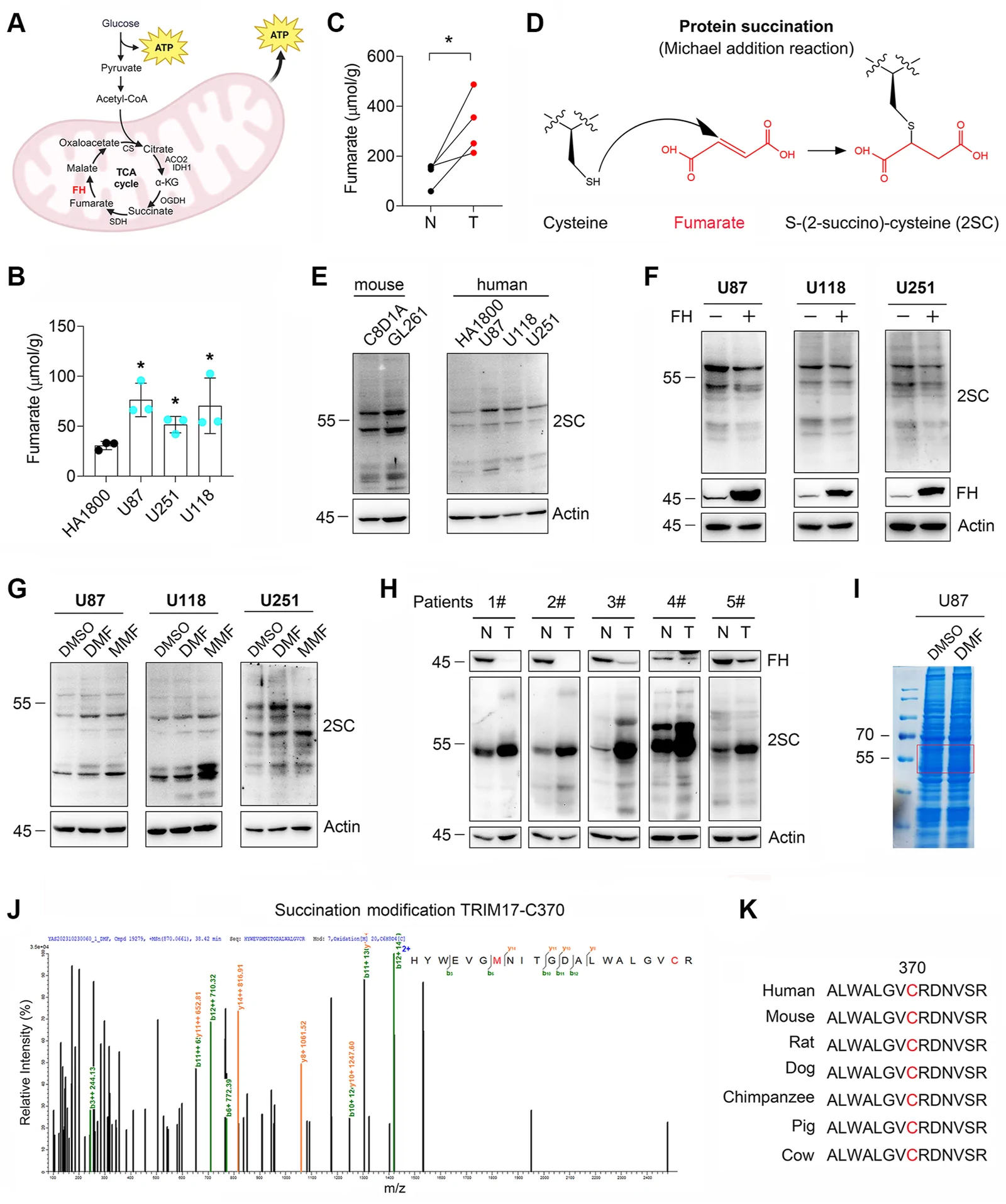
**FH loss drives fumarate accumulation and TRIM17 succination at Cys370 in GBM.***A*, schematic illustration of the mitochondrial TCA cycle highlighting fumarate generation. *B*, Fumarate levels are elevated in GBM cell lines (U87, U251, and U118) compared to normal human astrocytes (HA1800). *n* = *3* biologically independent replicates. *C*, fumarate accumulation is observed in glioma tissues compared with paired normal brain tissues. *n* = *4* biologically independent replicates. *D*, schematic representation of protein succination, where fumarate reacts with cysteine residues to form 2SC. *E*, elevated 2SC levels in GBM cell lines (U87, U118, U251, GL261) compared to normal human (HA1800) and mouse (C8D1A) astrocytes. *F*, FH overexpression reduces 2SC levels in GBM cells. Cells were transfected with a plasmid encoding *FH*. Twenty-four hours post-transfection, cells were lysed for Western blot analysis. *G*, treatment with fumarate derivatives MMF (250 μm) or DMF (10 μm) for 24 or 4 h increases 2SC levels in GBM cells, as shown by Western blot. *H*, clinical GBM tissues exhibit higher 2SC levels than paired normal brain tissues (*n* = 5). *I* and *J*, LC-MS/MS analysis identifies succination of TRIM17 at Cys370 in U87 cells treated with DMF (10 μm, 4 h). Whole-cell lysates were resolved by SDS-PAGE, stained with Coomassie Brilliant *Blue* and gel slices between 55 to 70 kDa were subjected to LC-MS/MS. *K*, sequence alignment of the Cys370 region in TRIM17 across multiple species. Data are presented as mean ± SD. Statistical significance was determined by unpaired Student’s *t* test (*B*) or paired Student’s *t* test (*C*). ∗*p* < 0.05. 2SC, S-(2-succino)-cysteine; FH, fumarate hydratase; GBM, glioblastoma; TRIM17, tripartite motif containing 17.

As FH catalyzes conversion of fumarate to malate in the TCA cycle, loss of FH activity may lead to intracellular fumarate accumulation (Fig. 3*A*). To explore the mechanism by which FH suppresses GBM malignancy, we first examined fumarate levels in GBM cells. Compared to normal human astrocytes (HA1800), fumarate levels were significantly elevated in U87, U251, and U118 cells (Fig. 3*B*). Conversely, FH overexpression decreased intracellular fumarate (Fig. S8*A*), while FH knockdown or pharmacological inhibition markedly increased fumarate accumulation (Fig. S8, *B* and *C*). Similarly, paired clinical glioma tissues showed elevated fumarate levels relative to adjacent normal tissues (Fig. 3*C*).

**Figure 4:**
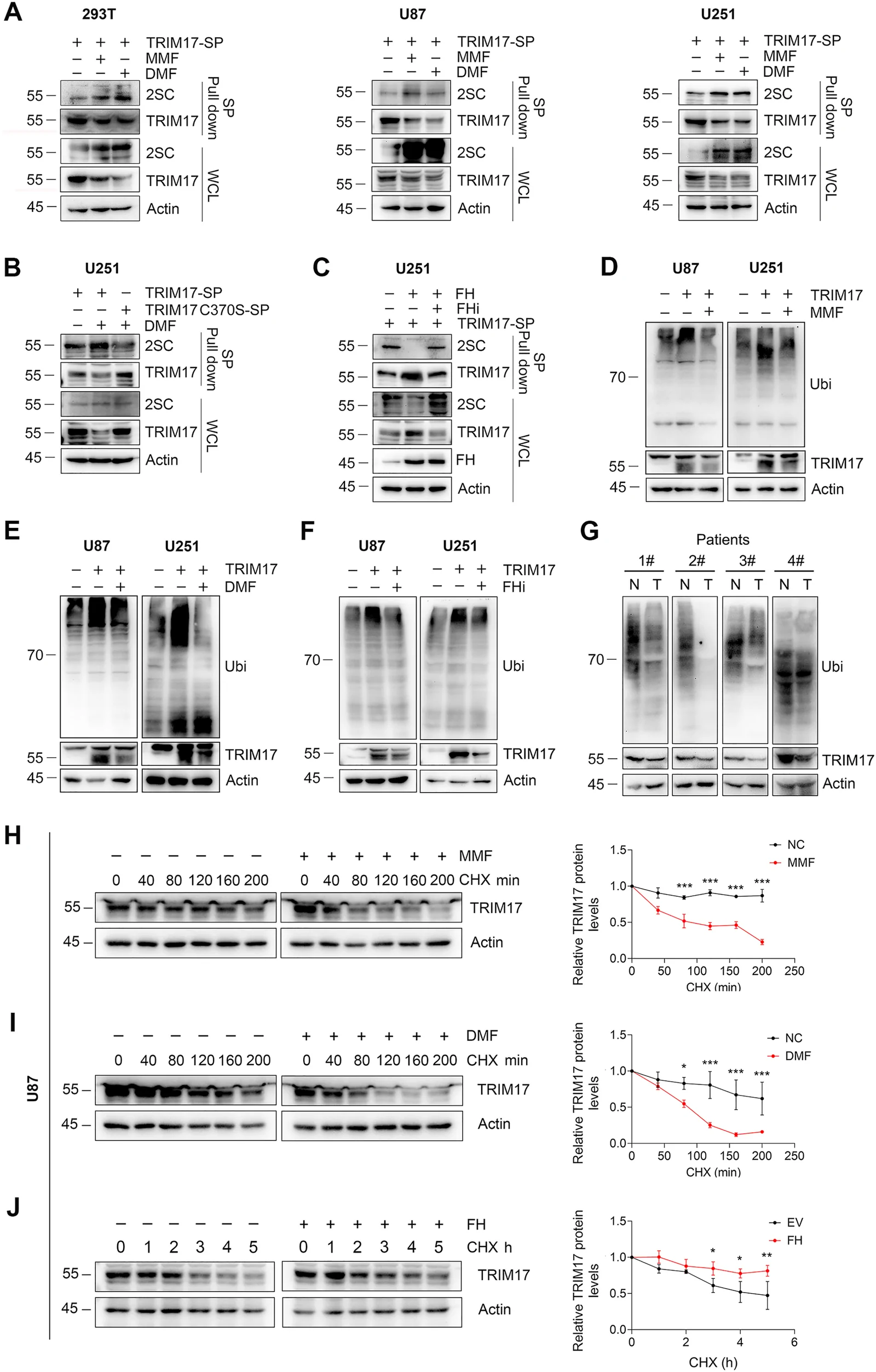
**Fumarate induces TRIM17 succination at Cys370, reducing its activity and stability.***A*, MMF and DMF induce succination of TRIM17. HEK293T, U87, and U251 cells were transfected with a plasmid expressing SP-tagged *TRIM17*. At 24 h post-transfection, cells were treated with MMF (250 μm, 24 h) or DMF (10 μm, 4 h), followed by SP pull-down and immunoblot analysis. *B*, mutation of Cys370 to Ser (C370S) abolishes DMF-induced succination. U251 cells were transfected with plasmids expressing SP-tagged WT *TRIM17* or *C370S TRIM17*, followed by DMF (10 μm, 4 h). *C*, FH overexpression decreases TRIM17 succination; this effect is reversed by FH inhibitor (10 μm, 18 h). U251 cells were cotransfected with SP-tagged *TRIM17* and/or *FH* plasmids. *D* and *E*, MMF and DMF treatments reduce global protein ubiquitination. Cell treatments as in (*A*). *F*, FH inhibition decreases global protein ubiquitination. Cell treatments as in (*C*). *G*, TRIM17 and global protein ubiquitination levels were reduced in GBM tumor tissues compared to adjacent normal tissues. *n* = 4. *H* and *I*, MMF and DMF destabilize TRIM17 protein. U87 cells were transfected with a plasmid expressing *TRIM17*. After 24 h, cells were treated with MMF (250 μm, 24 h) or DMF (10 μm, 4 h), followed by CHX, 50 μg/ml treatment. Cells were harvested at indicated time points for Western blot analysis. *n* = *3* biologically independent replicates. *J*, FH overexpression stabilizes TRIM17 protein under CHX treatment. Cell treatments as in (*H* and *I*). *n* = *3* biologically independent replicates. Protein levels were analyzed by Western blot using Actin as a loading control. N, normal brain tissues; SP, S-tagged protein; T, tumor brain tissues. Data are presented as mean ± SD. Statistical significance was determined by two-way ANOVA followed by Šidák’s *post hoc* test. ∗*p* < 0.05, ∗∗*p* < 0.01 and ∗∗∗*p* < 0.001. CHX, cycloheximide; FH, fumarate hydratase; GBM, glioblastoma; TRIM17, tripartite motif containing 17.

Fumarate acts as an endogenous electrophile that covalently modifies cysteine residues in proteins through a non-enzymatic Michael addition, forming 2SC adducts, a process known as protein succination (Fig. 3*D*). Western blot analysis revealed increased 2SC levels in both human (U87, U118, U251) and murine (GL261) GBM cell lines compared to normal astrocytes (Figs. 3*E* and S9*A*). FH overexpression reduced 2SC levels in U87, U118, and U251 cells, whereas treatment with fumarate derivatives (MMF/DMF) elevated 2SC accumulation (Figs. 3, *F*, *G* and S9, *B*, *C*). Of note, catalytically inactive FH mutants, FH D238H and FH E378K, had no effect on 2SC levels in U251 cells (Fig. S9*D*). Clinical glioma tissues displayed higher 2SC levels than paired adjacent normal tissues (Fig. 3*H*). These findings suggest that elevated fumarate levels in GBM induce protein succination, which may contribute to tumor progression. Notably, the succinated proteins predominantly appeared around 55 kDa on SDS-PAGE (Fig. 3, *E*–*H*). To identify these targets, we isolated ∼55 kDa protein bands from DMF-treated U87 cells and subjected them to LC-MS/MS analysis (Fig. 3*I*). Mass spectrometry identified 11 proteins with cysteine succination (Fig. S10*A*). Among them, tripartite motif containing 17 (TRIM17) stood out due to its known role in cancer progression and chemotherapy resistance (25, 26). Succination at cysteine (Cys370) of TRIM17 was detected in DMF-treated U87 cells (Fig. 3*J*), and sequence alignment revealed that Cys370 is highly conserved across species (Fig. 3*K*). Protein pull-down assays in HEK293T, U87, and U251 cells confirmed that MMF or DMF treatment increased TRIM17 succination (Fig. 4*A*). Mutation of Cys370 to serine (C370S) substantially reduced DMF-induced succination (Fig. 4*B*). Furthermore, FH overexpression suppressed TRIM17 succination, while FH inhibition restored it (Fig. 4*C*), further linking FH deficiency and fumarate accumulation to this post-translational modification. In addition, immunoprecipitation using an anti-TRIM17 antibody revealed that TRIM17 succination was reduced by FH overexpression, whereas DMF treatment increased TRIM17 succination (Fig. S10, *B* and *C*). Functional assays further showed that TRIM17 C370S-overexpressing cells were resistant to the FH knockdown-induced increases in cell viability, migration, and invasion (Fig. S11, *A*–*G*), indicating that TRIM17 succination at C370 plays an essential role in FH-mediated regulation of GBM cell growth and metastasis.

Given that TRIM17 functions as an E3 ubiquitin ligase (25, 27, 28). we next investigated whether succination at Cys370 affects its enzymatic activity. TRIM17 overexpression increased global protein ubiquitination in U87 and U251 cells; however, this effect was significantly attenuated by treatment with MMF or DMF (Fig. 4, *D* and *E*), suggesting that succinated TRIM17 loses its E3 ligase activity. Similarly, FH inhibition, as well as TRIM17 knockdown, suppressed TRIM17-induced ubiquitination (Figs. 4*F* and S12, *A*, *B*). Consistent with these findings, clinical glioma samples exhibited decreased TRIM17 expression and reduced global ubiquitination (Fig. 4*G*). More specifically, we examined the ubiquitination of MCL1, a well-established substrate of TRIM17, to further assess TRIM17 enzymatic activity (29). Our results showed that TRIM17 overexpression significantly increased the ubiquitination of MCL1, whereas treatment with DMF partially reversed this effect (Fig. S9*E*). These findings further support the notion that DMF-mediated succination impairs the E3 ubiquitin ligase activity of TRIM17. In addition, we also observed that TRIM17 protein levels were decreased following MMF or DMF treatment, while its expression was increased in FH-overexpressing cells, implying that succination at Cys370 may affect TRIM17 protein stability. To test this, we performed cycloheximide chase assays. Both MMF and DMF treatment significantly reduced TRIM17 stability in U87 and U251 cells (Figs. 4, *H*, *I* and S13, *A*, *B*), whereas FH overexpression enhanced TRIM17 protein stability (Figs. 4*J* and S13*C*). To examine the degradation pathways of TRIM17, we used MG132 and chloroquine (CQ) to inhibit the proteasomal and lysosomal pathways, respectively. The results showed that DMF treatment markedly decreased TRIM17 protein levels, which were largely rescued by both MG132 and CQ (Fig. S13*D*), indicating that TRIM17 degradation is likely mediated by both the proteasome and lysosome pathways.

**Figure 5:**
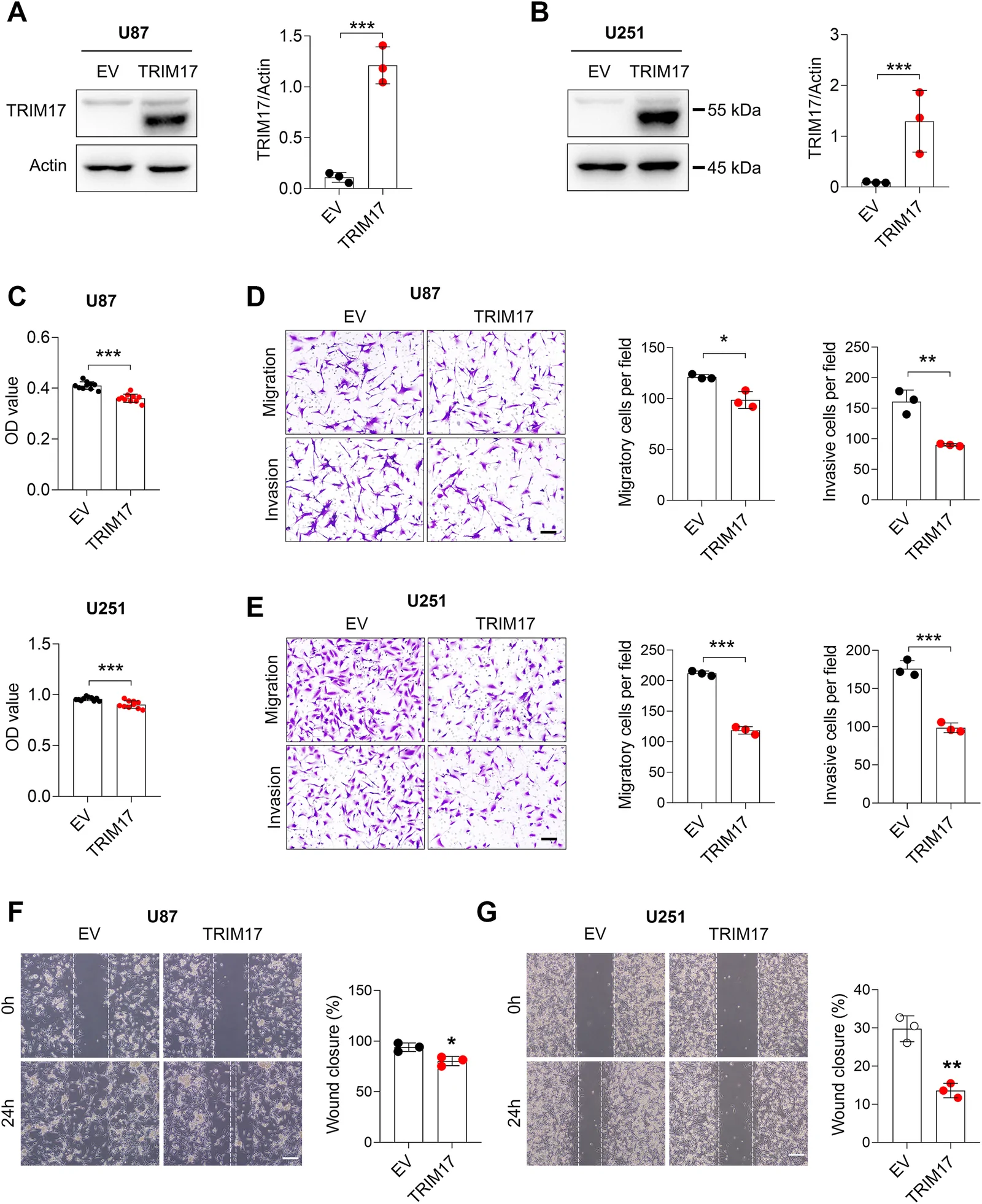
**TRIM17 suppresses GBM cell proliferation, migration, and invasion.***A* and *B*, Western blot analysis confirming TRIM17 overexpression in GBM cells. U87 and U251 cells were transfected with a plasmid expressing *TRIM17* for 24 h. Actin was used as a loading control. *n* = 3 biologically independent replicates. *C*, TRIM17 overexpression reduces U87 and U251 cell viability. Cells were transfected with a plasmid expressing *TRIM17* for 24 h *n* = *10* biologically independent replicates per group. *D* and *G*, TRIM17 overexpression inhibits U87 and U251 cell migration and invasion, as shown by transwell (*D* and *E*, the scale bar represents50 μm) and wound healing (*F* and *G*, this scale bar represents100 μm). *n* = *3* biologically independent replicates. Data are presented as mean ± SD. Statistical significance was determined by unpaired Student’s *t* test. ∗*p* < 0.05, ∗∗*p* < 0.01 *and* ∗∗∗*p* < 0.001. TRIM17, tripartite motif containing 17.

Next, we investigated whether TRIM17 influences GBM cell growth and metastasis. To this end, we overexpressed TRIM17 in U87 and U251 cells (Fig. 5, *A* and *B*). Functional assays showed that TRIM17 overexpression reduced GBM cell viability, migration, and invasion (Fig. 5, *C*–*G*). On the contrary, TRIM17 knockdown enhanced GBM cell viability, migration, and invasion (Fig. S14, *A*–*E*), suggesting that TRIM17 functions as a negative regulator of GBM malignancy. Moreover, TRIM17 knockdown abolished FH-induced decreases in cell viability, migration, and invasion in U251 cells (Fig. S15, *A*–*G*). To further support this notion, we analyzed TRIM17 expression using publicly available datasets. CGGA data revealed a progressive decline in TRIM17 expression with increasing tumor grade (Fig. S16*A*). Kaplan-Meier survival analyses showed that low TRIM17 expression correlated with shorter OS across multiple glioma cohorts (Fig. S16, *B*–*D*), supporting a tumor-suppressive role for TRIM17 in GBM. Collectively, these findings indicate that FH suppresses GBM malignancy, at least in part, through fumarate-mediated succination of TRIM17 at Cys370, thereby impairing its tumor-suppressive function.

### FH suppresses GBM progression *in vivo*

**Figure 6:**
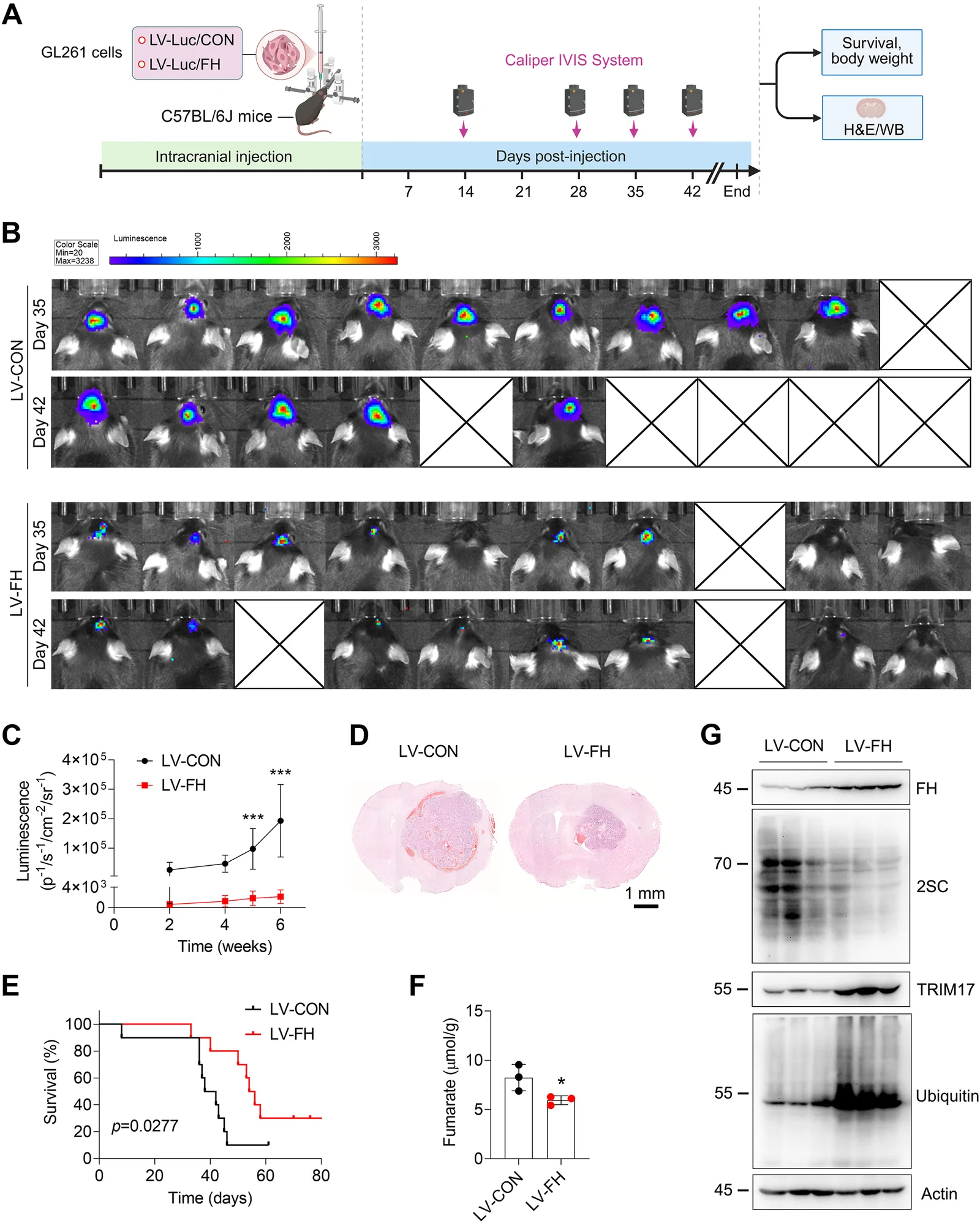
**FH overexpression suppresses tumor growth in an orthotopic xenograft model.***A*, schematic diagram of the experimental workflow. GL261 cells were transduced with lentivirus expressing luciferase and FH or control virus. After 48 h, transduced cells were selected using puromycin (2.5 μg/ml), and then injected intracranially into C57BL/6J mice. *B*, representative bioluminescence images of mice bearing GL261 orthotopic xenografts at the indicated time points. *n* = 10 biologically independent animals. *C*, quantification of bioluminescence signal intensity. *n* = 10 biologically independent animals. *D*, *H*&E staining of representative brain sections showing intracranial tumors. This scale bar represents1.0 mm. *E*, Kaplan-Meier survival analysis indicates prolonged survival in the LV-FH group. *F*, fumarate levels were reduced in LV-FH tumors. *n* = 3 biologically independent animals. *G*, FH, 2SC, TRIM17, and Ubiquitin protein levels in orthotopic xenografts. *n* = 3 biologically independent animals. Protein levels were analyzed by Western blot using Actin as a loading control. Data are presented as mean ± SD. Statistical significance was determined by two-way ANOVA followed by Šidák’s *post hoc* test (*C*), log-rank test (*E*) and unpaired Student’s *t* test (*F*). ∗*p* < 0.05, ∗∗*p* < 0.01 and ∗∗∗*p* < 0.001. FH, fumarate hydratase; TRIM17, tripartite motif containing 17.

To validate our findings *in vivo*, we established orthotopic xenograft models using GL261 cells (Fig. 6*A*). Mice were injected intracranially with GL261 cells transduced with either luciferase/FH-overexpressing lentivirus FH (LV-Luc/FH) or control virus (LV-Luc/CON) (Fig. S17*A*). Bioluminescence imaging revealed a marked reduction in tumor growth in the FH-overexpression group (Figs. 6*B* and S17*B*). The quantification of bioluminescence signal intensity confirmed the reduced tumor burden in the FH overexpression group (Fig. 6*C*). H&E staining of brain sections showed smaller, less invasive tumors in the FH-overexpression group (Fig. 6*D*). Consistent with these observations, Kaplan-Meier survival analysis indicated that FH overexpression significantly prolonged survival in tumor-bearing mice (Fig. 6*E*), without affecting body weight (Fig. S17*C*). Metabolically, FH overexpression reduced intratumoral fumarate levels (Fig. 6*F*). Western blot analysis further showed increased TRIM17 expression and global ubiquitination, along with reduced 2SC level in tumors from the FH-overexpression mice (Fig. 6*G*). Together, these results indicate that FH overexpression restores TRIM17 stability and activity by limiting fumarate-induced succination, thereby suppressing GBM growth *in vivo*.

**Figure 7:**
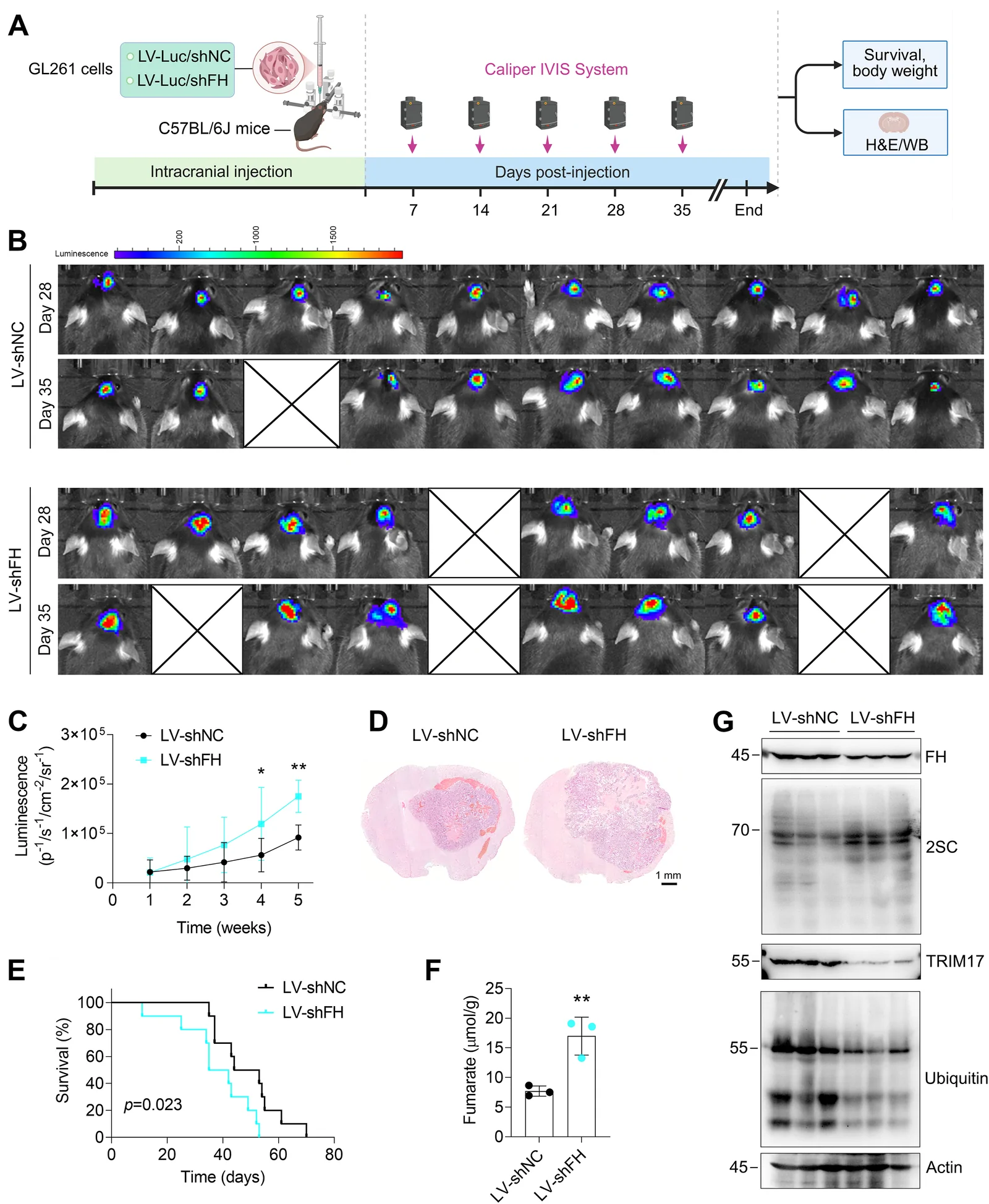
**FH knockdown promotes tumor growth in an orthotopic xenograft model.***A*, schematic overview of the experimental workflow. GL261 cells were transduced with lentivirus expressing luciferase and FH-targeting shRNA or control virus. After 48 h, transduced cells were selected using puromycin (2.5 μg/ml), and then injected intracranially into C57BL/6J mice. *B*, representative *in vivo* bioluminescence images of orthotopic xenograft-bearing mice at indicated time points. *n* = 10 biologically independent animals. *C*, quantification of bioluminescence signal intensity. *n* = 10 biologically independent animals. *D*, representative H&E-stained brain sections showing xenograft tumors. The scale bar represents 1.0 mm. *E*, Kaplan-Meier survival analysis showing reduced survival in the lentivirus carrying *FH*-targeting shRNA group. *F*, fumarate levels were increased in tumors from the lentivirus carrying *FH*-targeting shRNA group. *n* = 3 biologically independent animals. *G*, FH, 2SC, TRIM17, and Ubi protein levels in orthotopic xenografts. *n* = 3 biologically independent animals. Protein levels were analyzed by Western blot using Actin as a loading control. Data are presented as mean ± SD. Statistical significance was determined by two-way ANOVA followed by Šidák’s *post hoc* test (*C*), log-rank test (*E*) and unpaired Student’s *t* test (*F*). *∗p* < 0.05, *∗∗p* < 0.01 and ∗∗∗*p* < 0.001. FH, fumarate hydratase; TRIM17, tripartite motif containing 17; Ubi, ubiquitin.

To further validate these findings, we performed FH knockdown in orthotopic xenograft models (Fig. 7*A*). FH knockdown in GL261 cells was achieved using a lentivirus carrying *FH*-targeting shRNA (Fig. S18*A*). Bioluminescent imaging showed significantly enhanced tumor growth in the FH-silence group (Figs. 7, *B*, *C*, and S18*B*). H&E staining confirmed the presence of large and more infiltrative tumors in mice bearing FH-knockdown xenografts (Fig. 7*D*). Consistently, OS was reduced in mice with lentivirus carrying *FH*-targeting shRNA-treated cells (Fig. 7*E*), while body weight remained unaffected (Fig. S18*C*). Metabolically, FH knockdown led to elevated fumarate accumulation in xenografts (Fig. 7*F*). Western blot analysis showed reduced expression of TRIM17 and ubiquitin, alongside increased 2SC levels (Fig. 7*G*), indicating that loss of FH enhances fumarate-induced TRIM17 succination and compromises its E3 ubiquitin ligase activity. Collectively, these findings further confirm that FH suppresses GBM progression by limiting fumarate accumulation and preventing pathological TRIM17 succination, thereby preserving TRIM17 stability and tumor-suppressive function *in vivo*.

## Discussion

Metabolic reprogramming has emerged as a fundamental hallmark of cancer, enabling tumor cells to adapt to adverse microenvironments and sustain uncontrolled proliferation (5, 30, 31, 32, 33, 34, 35). In GBM, mitochondrial dysfunction and TCA cycle alterations are frequently observed (12, 13, 14, 36). However, the contribution of specific metabolic enzymes to GBM pathogenesis remains incompletely understood. Here, we identify FH as a tumor suppressor in glioma and uncover a novel mechanism linking fumarate accumulation to TRIM17 succination, ultimately promoting TRIM17 degradation and thereby triggering tumor progression.

Our data demonstrate that both FH protein and mRNA levels are markedly downregulated in GBM tissues compared to normal brain and that lower FH levels correlate with poor patient prognosis. Functional assays confirmed the tumor-suppressive role of FH in GBM, revealing its impact on cellular proliferation, migration, and survival. These results are consistent with previous studies implicating FH loss in tumorigenesis across multiple cancer types, including renal cell carcinoma and uterine leiomyomas (37, 38). Currently, the precise mechanism underlying FH downregulation in GBM remains unclear. This downregulation may result from genetic alterations, epigenetic regulation, or post-transcriptional regulation. As a result, FH deficiency leads to intracellular accumulation of fumarate, which acts as an oncometabolite by covalently modifying cysteine residues *via* 2SC adduct formation, a process termed succination (20). Elevated 2SC levels have been observed in fumarate hydratase-deficient tumors and are increasingly recognized as biomarkers of metabolic dysregulation (21, 23). In GBM cells, we found that fumarate accumulation promotes global protein succination, with the E3 ubiquitin ligase TRIM17 identified as a key succinated target. Interestingly, while previous studies have highlighted the oncogenic role of fumarate accumulation through epigenetic reprogramming, such as inhibition of α-ketoglutarate-dependent dioxygenases (39), our work reveals an additional layer of regulation involving post-translational modification of E3 ligases. These findings suggest that fumarate-mediated succination exerts broad effects on protein homeostasis, beyond canonical transcriptional regulation.

Succination of TRIM17 at Cys370 impairs its E3 ligase activity, as evidenced by reduced global protein ubiquitination in FH-silent and fumarate-treated GBM cells. These findings extend recent observations that metabolic alterations can directly modulate the ubiquitin-proteasome system, contributing to oncogenic processes (40, 41). Notably, this succination modification reduces TRIM17 protein stability. In line with our findings, previous studies have shown that succination can alter protein function and stability in cancer. For instance, KEAP1 succination inactivates its ability to degrade NRF2, resulting in constitutive activation of antioxidant responses (42). Similarly, PTEN succination at C211 disrupts its tumor-suppressive activity, leading to PI3K/AKT signaling activation in renal cancer (24) and promoting glioma stem cell maintenance *via* iron-sulfur cluster assembly (21), ZAP70 succination has also been shown to impair CD8^+^ T cell activation, enabling immune evasion (22). Importantly, we observed that TRIM17 is significantly downregulated in glioma patient samples and its low expression correlates with poor survival, reinforcing its role as a tumor suppressor in GBM. Our data further support this, indicating that TRIM17 overexpression reduces GBM cell viability and metastasis, which is likely due to enhanced pro-apoptotic activity (27, 29). Interestingly, TRIM17 may function in a context-dependent manner. While it acts as a tumor suppressor in GBM, a recent study reported that TRIM17 promotes gastric cancer progression by regulating BAX protein stability and antagonizing apoptosis (25). The basis for this discrepancy remains unclear but may reflect tumor-specific regulatory networks. Moreover, our analysis revealed that high TRIM17 expression correlates with increased chemotherapy sensitivity in GBM. These contracts with a report showing that TRIM17 promotes cisplatin resistance in nonsmall cell lung cancer (26), again underscoring the tissue- and context-specific functions of TRIM17. From a therapeutic perspective, strategies aimed at reducing fumarate accumulation, restoring FH activity, or pharmacologically reactivating succinated E3 ligases such as TRIM17 could provide novel approaches for GBM treatment.

Notably, it should be noted that, as an E3 ubiquitin ligase, TRIM17 is unlikely to function as the terminal effector mediating its tumor-suppressive effects in GBM. Rather, TRIM17 is expected to exert its biological functions through the ubiquitination and regulation of downstream substrate proteins. To further elucidate the molecular mechanisms underlying TRIM17-mediated tumor suppression, we are currently conducting studies aimed at identifying and characterizing its downstream substrates. Additionally, we assessed cell viability using the 3-(4,5-dimethylthiazol-2-yl)-2,5-diphenyltetrazolium bromide (MTT) assay following FH overexpression or knockdown. Because FH is a mitochondrial enzyme, altering its expression may affect mitochondrial metabolic activity, which could potentially influence the readout of the MTT assay independently of changes in cell viability. In future studies, we will further validate our findings using complementary approaches, such as the CCK-8 assay, flow cytometric analysis, and trypan blue exclusion, to provide a more comprehensive assessment of cell viability.

In conclusion, our study identifies a previously unrecognized FH-TRIM17 axis that mechanistically links metabolic dysregulation to proteostasis in GBM. We provide compelling evidence that FH deficiency leads to fumarate accumulation, which impairs TRIM17 E3 ligase activity through succination, resulting in stabilization of oncogenic substrates and promotion of tumor progression. These findings demonstrate that dysfunction of the TCA cycle contributes to GBM pathogenesis not only by metabolic rewiring but also by disrupting ubiquitin-proteasome-mediated protein degradation. Collectively, our work highlights a novel metabolic–proteostatic signaling pathway in GBM with potential implications for targeted therapeutic intervention.

## Experimental procedures

### Antibodies and reagents

Rabbit polyclonal anti-FH/fumarase (ab233394) was purchased from Abcam (Cambridge, UK). Mouse monoclonal antibodies against Ubiquitin (P4D1, 3936S) and Actin (8H10D10, 3700) were purchased from Cell Signaling Technology. Rabbit polyclonal anti-2SC (crb2005017d/6774) was sourced from Discovery Antibodies (CRB). Rabbit polyclonal anti-TRIM17 (#13663-1-AP), mouse monoclonal anti-TOM20 (#66777-1-Ig), rabbit polyclonal anti-MCL1 (#16225-1-AP), and light-chain specific HRP-conjugated IgG fraction monoclonal mouse anti-Rabbit IgG (#SA00001-7L) were acquired from Proteintech. Rabbit anti-Flag (F1804) were purchased from Sigma Sigma-Aldrich. HRP-conjugated anti-mouse and anti-rabbit secondary antibodies were purchased from Jackson ImmunoResearch. S-protein agarose (#69704) was obtained from Millipore (Billerica). Myc-Tag (Sepharose Bead Conjugate) (#20168) was obtained from Thermo Fisher Scientific (Waltham). FH inhibitor, Fumarate hydratase-IN-1 (HY-100004) was purchased from MedChemExpress. The cell-membrane-permeable derivative of fumarate, mono-Methyl fumarate (#651419) and Dimethyl fumarate (#242926), were purchased from Sigma-Aldrich.

### Cell culture

Human GBM cell lines U87, U251, and U118, normal human astrocyte cell line HA1800, mouse GBM cell line GL261, and normal mouse astrocyte cell line C8D1A were obtained from Shanghai Cell Bank (Shanghai Biological Sciences, Chinese Academy of Sciences). HEK293T cells were acquired from the American Type Culture Collection (ATCC). All cells were maintained in Dulbecco’s Modified Eagle’s Medium (DMEM) supplemented with 10% fetal bovine serum (FBS; Gibco), 100 U/ml penicillin, and 100 μg/ml streptomycin (Life Technologies) at 37 °C in a humidified incubator with 5% CO_2_, except for U87 cells, which were cultured in α-MEM medium (Hyclone) with the same supplements as mentioned above. All cell lines were authenticated by short tandem repeat polymorphism and underwent routine *mycoplasma* testing by PCR.

### Human GBM tumor specimens

Human GBM tumor tissues employed for Western blot, IHC, and qRT-PCR analyses were procured from the Affiliated Hospital of Nantong University. Patients #1, #4, and #5 harbored isocitrate dehydrogenase-mutant tumors, whereas patients #2 and #3 had isocitrate dehydrogenase -WT tumors. All freshly obtained tissues were gathered subsequent to surgical resection, promptly rinsed with sterile phosphate-buffered saline (PBS), and instantaneously fixed in 4% paraformaldehyde (PFA) for 12 h prior to being embedded in paraffin or stored at −80 °C. Written informed consent from patients was acquired prior to the initiation of the study. Human sample collection for research was conducted in accordance with the recognized ethical guideline of Declaration of Helsinki and approved by the Ethics Committee of Affiliated Hospital of Nantong University (No. 2022-L125).

### Plasmid construction

The full-length coding regions of *FH and TRIM17* were synthesized by Heyuan Biotechnology Company. The synthesized *FH* and *TRIM17* were cloned into pcDNA3.1(+) vector (Invitrogen) using Nhe I and BamH I restriction sites. All correct constructions were confirmed by sequencing. Site-directed mutagenesis was performed to generate FH D238H, FH E378K, siRNA-resistant FH, and TRIM17 C370S using a PCR-based mutagenesis kit (Stratagene). Primer sequence used for mutagenesis is provided in Table S1. The mutant plasmid was confirmed by sequencing. For the transient transfections, plasmids were transfected into GBM cells with Lipofectamine 2000 (Invitrogen), following the manufacturer's instructions.

For siRNA-mediated gene knockdown, *FH*-specific siRNAs and a negative control siRNA were chemically synthesized (RiboBio). The siRNA sequences used in this study are listed in Table S1. The siRNAs were transfected into GBM cells with Lipofectamine RNAiMAX transfection reagent (Invitrogen) according to the manufacturer’s instructions.

### qRT-PCR

To investigate the expression levels of target genes in GBM cells, qRT-PCR was performed. Total RNA was extracted from the GBM cells using TRIzol reagent (Invitrogen, Carlsbad, CA), followed by quantification using spectrophotometry. The extracted RNA was then reversely transcribed into the first strand cDNA using the PrimeScript RT reagent kit (Takara) according to the manufacturer’s protocol. qRT-PCR amplification was carried out using the Fast Start Universal SYBR Green Master Mix (Roche on a Roche LightCycler 96 system. The thermal cycling conditions were set as follows: initial denaturation at 95 °C for 6 min, followed by 40 cycles of denaturation at 95 °C for 10 s, annealing at 60 °C for 30 s, and extension at 72 °C for 10 s. Primer sequences used in the experiment are provided in Table S2. Relative quantitation of target genes was normalized to that of *18S rRNA*, and were analyzed using the 2^−△△CT^ method (43).

### Protein extraction and Western blot analysis

Western blot assay was performed as previously described (44). Briefly, Total protein was extracted from cells and tissues using protein lysis buffer supplemented with protease and phosphatase inhibitors (Roche Applied Science). Protein concentrations were assayed by the Pierce BCA Protein Assay Kit (Thermo Fisher Scientific). Subsequently, equal amounts of protein (50 μg) were separated by 8% or 10% SDS-PAGE and transferred onto 0.45 μm PVDF membranes. After blocked with 5% BSA in TBST for 1 h, the membranes were incubated at 4 °C overnight with the following primary antibodies: rabbit anti-FH/fumarase, mouse anti-Ubiquitin, mouse anti-Actin, rabbit anti-2SC, rabbit anti-TRIM17, and rabbit anti-MCL1. Thereafter, the membranes were incubated with HRP-conjugated secondary antibodies for 1 h. Protein bands were visualized using an enhanced chemiluminescence reagent (Thermo Fisher Scientific) and quantitative analyzed using ImageJ (https://imagej.net/ij/) software (NIH).

### Co-immunoprecipitation (Co-IP) and ubiquitination assay

Cells were transfected with either the TRIM17 overexpression plasmid or an empty vector control and subsequently treated with or without DMF (10 μm). Cells were lysed in ice-cold lysis buffer supplemented with protease and deubiquitinase inhibitors (Roche Applied Science). After centrifugation to remove cellular debris, a portion of the lysate was collected as the input control. The remaining lysate was incubated overnight at 4 °C with an anti-MCL1 antibody, followed by incubation with Protein A/G agarose beads (#20421, Thermo Fisher Scientific) to immunoprecipitate MCL1-containing protein complexes. The beads were then washed extensively to remove nonspecifically bound proteins, and the immunoprecipitated proteins were eluted by boiling in 2 × SDS sample loading buffer. Both input and immunoprecipitated samples were separated by SDS-PAGE and subjected to immunoblotting with antibodies against ubiquitin, TRIM17, and MCL1. Actin was detected in the input samples as a loading control to verify equal protein loading.

### Cell viability assay

Cell viability was evaluated using the MTT) assay (S19063) (45). Briefly, cells were seeded into 96-well plates at a density of 2 × 10^3^ cells per well in triplicate and allowed to adhere overnight. Following transfection with plasmids or siRNAs, cells were incubated for 24, 48, and 72 h. At each time point, 10 μl of MTT solution (5 mg/ml in PBS) was added to each well, and the plates were incubated at 37 °C for 4 h in a humidified 5% CO_2_ atmosphere. Subsequently, 100 μl of 20% SDS solution was added to dissolve the formazan crystals. The absorbance was measured at 570 nm using a microplate reader (BioTek). The results were expressed as the mean optical density values, reflecting the relative number of viable cells at each time point.

### Colony-forming assay

Cells in the logarithmic growth phase were harvested by trypsinization and resuspended in culture medium to obtain a single-cell suspension. After viable cell counting, cells were seeded uniformly into 6-well plates at an appropriate density (300 cells per well). Subsequently, cells were incubated continuously at 37 °C in a humidified incubator with 5% CO_2_. The culture medium was refreshed every 3 days. After 14 days of culture, when macroscopic colonies were clearly visible, the culture was terminated. The cells were gently washed twice with pre-chilled PBS, fixed with 4% paraformaldehyde solution for 15 min at room temperature, and stained with 0.1% crystal violet solution for 30 min. Excess dye was slowly rinsed off with running distilled water, and the plates were air-dried naturally at room temperature. Colonies containing more than 50 viable cells were counted under a light microscope. The colony formation rate was calculated according to the following formula: Colonyformationrate(%)=((Numberofformedcolonies)/(Numberofseededcells))×100% (46).

### Wound healing assay

The wound healing assay was conducted to evaluate cell migration capabilities using ibidi Culture Inserts (ibidi GmbH) to ensure consistent initial wound sizes (47). Briefly, 3 × 10^4^ cells were seeded into the inserts (70 μl cell suspension per chamber) and cultured in medium supplemented with 10% FBS. After 24 h, when cells reached approximately 100% confluence, the inserts were carefully removed to create uniform wounds. The wells were gently washed with PBS to eliminate detached cells. Cells were then maintained in DMEM, and images of the wounds were captured at 0 h and 24 h post-wounding. The wound closure rate was quantified using ImageJ software with the following formula: Woundhealingrate(%)=[(Woundareaat0h−Woundareaat24h)/Woundareaat0h]×100%

### Transwell assay

Cell migration and invasion were assessed using a transwell assay system. For migration analysis, 4 × 10^4^ cells suspended in 200 μl of serum-free medium were seeded into the upper chambers of transwell inserts (8.0 μm pore size) in 24-well plates. For invasion evaluation, the upper chambers were pre-coated with 80 μl of Matrigel (BD Bioscience), and 5 × 10^4^ cells in 200 μl of serum-free medium were added. The lower chambers were filled with 500 μl of DMEM containing 10% FBS as a chemoattractant. Following 24 h of incubation at 37 °C, nonmigrating or noninvading cells on the upper surface of the membrane were carefully removed using cotton swabs. The membranes were then fixed with 4% PFA for 20 min and stained with 0.1% crystal violet for 30 min. Migrated and invaded cells on the lower surface of the membrane were visualized and quantified by counting three randomly selected fields under a light microscope (Olympus BX51) at 200× magnification.

### Protein pull-down

Protein pull-down was performed using a previously reported method (48). Briefly, cells were transfected with plasmids expressing *TRIM17* with S-protein tag. After 24 h, cells were harvested, and total cell lysates were prepared. The lysates were incubated with S-tag magnetic beads (#69704, Millipore) at 4 °C for 4 h on a rotator to facilitate binding. Unbound proteins were removed by washing the beads with ice-cold wash buffer. Bound proteins were eluted and denatured by adding 2 × SDS loading buffer, followed by boiling at 100 °C for 5 min. After cooling to room temperature, the samples were subjected to Western blot analysis. After coomassie-blue or silver-nitrate staining, bands with interested proteins were excised for LC-MS/MS spectrometry analysis.

### LC-MS/MS analysis

LC-MS/MS analysis was conducted at Shanghai Applied Protein Technology. Eluted protein complexes from the pull-down assay were subjected to in-solution digestion and LC-MS/MS analysis to identify interacting partners. Briefly, proteins were reduced, alkylated, and enzymatically digested with trypsin (Promega) at a 1:50 enzyme-to-protein ratio for 20 h at 37 °C. Digested peptides were desalted, lyophilized, and reconstituted in 0.1% formic acid. Peptides were separated using an Easy-nLC 1000 ultra-high-performance liquid chromatography system (Thermo Fisher Scientific) and analyzed on a Q Exactive mass spectrometer (Thermo Fisher Scientific) operating in positive ion mode. The mobile phases consisted of 0.1% formic acid in water (A) and 84% acetonitrile with 0.1% formic acid (B). Full MS scans were acquired in the m/z range of 300 to 1800 with a resolution of 70,000 at m/z 200, followed by data-dependent MS/MS acquisition of the top 20 most intense ions using higher-energy collisional dissociation at 27 eV with a resolution of 17,500.

Raw data files were analyzed using MaxQuant software (https://www.maxquant.org/) (version 1.6.14) or Proteome Discoverer software (https://www.thermofisher.com/us/en/home/industrial/mass-spectrometry/liquid-chromatography-mass-spectrometry-lc-ms/lc-ms-software/multi-omics-data-analysis/proteome-discoverer-software.html) (version 2.5, Thermo Fisher Scientific) and searched against the UniProt *Homo sapiens* database using standard parameters. The search parameters included chymotrypsin digestion allowing up to two missed cleavage, fixed modifications of carbamidomethyl (Cys, C_2_H_3_NO, + 57.02 Da), variable modifications of oxidation (Met, O, + 15.99 Da), succination (Cys, C_4_H_4_O_4_, +116.01 Da), monomethyl succination (Cys, C5H6O4, + 130.03 Da), dimethyl succination (Cys, C_6_H_8_O_4_, + 144.04 Da) and ubiquitination on lysine residues (Lys, C_8_H_12_N_2_O_3_, + 114.04 Da). Precursor peptide mass tolerance was set to 0.01 Da, and MS/MS tolerance was set to 0.1 Da. The false discovery rate was set to ≤1% at both the peptide and protein levels.

### Immunofluorescence

To observe the subcellular localization of transfected FH protein, cells were seeded onto glass coverslips in 24-well plates at a density of 1 × 10^4^ cells per well. The following day, cells were transfected with a plasmid expressing FH-flag. After 24 h, cells were fixed with methanol at room temperature for 15 to 20 min and washed three times with PBS for 10 min each. Cells were then blocked with 5% BSA containing 0.1 to 0.5% Triton X-100 for 2 h at room temperature. Following blocking, cells were incubated overnight at 4 °C with primary antibodies, including rabbit anti-Flag and mouse anti-TOM20. After primary antibody incubation, cells were washed and incubated with Alexa Fluor 488/594-conjugated secondary antibodies for 2 h at room temperature. Nuclei were counterstained with DAPI. Fluorescence images were captured at 400× magnification using an Olympus fluorescence microscope (BX51).

### Immunohistochemistry

Immunohistochemical staining was performed as previously described (49). Briefly, 4% paraformaldehyde-fixed tissue sections were subjected to deparaffinization in xylene for 10 min three times, followed by rehydration in graded alcohols (100%, 100%, 95%, 90%, 80%, and 70%, each for 5 min), and ultimately washed with 0.1 M PBS three times. The slides were immersed in Tris-EDTA buffer (100 °C, 20 min) for antigen retrieval, and allowed to cool naturally at room temperature. After being treated with endogenous peroxidase blocker buffer for 5 min, blocking was carried out with 10% goat serum (containing 0.1% triton-100) for 30 min at 37 °C, and then incubated with primary antibodies against FH overnight at 4 °C. After washing with 0.1 M PBS three times, the sections were incubated with HRP-conjugated secondary antibodies for 1 h at room temperature. For visualization, freshly prepared DAB substrate (HC301; Vazyme) (1 ml DAB Buffer + 100 μl 10 × DAB Solution) was applied to the sections. The reaction was carefully monitored under a microscope for 1 to 5 min to achieve optimal staining intensity, followed by a 10 min wash with distilled water to terminate the reaction. Subsequently, hematoxylin was employed for redyeing, and finally the sections were dehydrated in graded alcohols and made transparent in xylene. Stained sections were imaged under a light microscope, and five random fields per section were captured at 100× and 400× magnification. Quantitative analysis was performed using Image-Pro Plus 6.0 software (https://www.mediacy.com/imageproplus) (Media Cybernetics). The area indicates the total area of the stained region; the IOD represents the total optical density of the brown-stained areas; integral optical density /area represent the expression quantification of the target protein. The signal density of the stained regions from five randomly selected fields was quantified in a blinded manner and subjected to statistical analysis.

### H&E staining

H&E staining was performed according to a previous protocol (50). Briefly, freshly dissected brains were fixed in 4% PFA for 24 h at 4 °C. After gradient dehydration in sucrose solutions (20% and 30% in 0.1 M PB), brains were embedded in optimal cutting temperaturecompound (Sakura Finetek) and rapidly frozen at −20 °C. Coronal sections (12 μm thick) were cut using a cryostat (Leica CM3050S, at −20 °C and mounted onto Superfrost Plus slides (Thermo Fisher Scientific). Frozen brain sections were rinsed in PBS three times for 5 min each. Nuclei were stained with hematoxylin at room temperature for 10 min, followed by bluing under running tap water. Sections were then differentiated in 1% hydrochloric acid in ethanol for 3 s and rinsed again with tap water. Cytoplasmic staining was carried out with eosin at room temperature for 30 s. The sections were then dehydrated sequentially in 95% ethanol (twice for 5 min), 100% ethanol (twice for 5 min), 100% ethanol/xylene (1:1, 5 min), and xylene (twice for 5 min), and finally coverslipped with neutral balsam. Stained sections were examined under a light microscope (Carl Zeiss AG).

### Cycloheximide chase assay

To determine protein stability, U251 cells were treated with CHX (50 μg/ml) to inhibit protein synthesis, and samples were collected at different time points as indicated. Cells were lysed in RIPA buffer, and protein concentrations were quantified using the BCA assay. Equal amounts of protein were separated by SDS-PAGE, transferred to PVDF membranes, and probed with anti-TRIM17, and anti-Actin antibodies. Band intensities were quantified using ImageJ software.

### Fumarate quantification

Fumarate levels in tissues and cells were quantified using a Fumarate Assay Kit (MAK060, Sigma-Aldrich) according to the manufacturer’s instructions. Briefly, tissues (40 mg) or cells (1 × 10^6^) were rapidly homogenized in 100 μl of Fumarate Assay Buffer. The homogenates were centrifuged at 13,000*g* for 10 min at 4 °C to remove insoluble material. Subsequently, 50 μl of the supernatant was transferred in duplicate to a 96-well plate. A master reaction mix was prepared according to the kit protocol, and 100 μl of the mix was added to each well. The plate was gently mixed using a horizontal shaker or by pipetting and incubated at room temperature for 30 min. Absorbance was measured at 450 nm using a microplate reader (BioTek). To account for background signal, the absorbance value of the blank (0 μm fumarate standard) was subtracted from all sample readings. A standard curve was generated using the absorbance values of the provided fumarate standards. The fumarate concentration in each sample was determined by interpolating the corrected absorbance values against the standard curve.

### Lentivirus transduction

LV-Luc/FH shRNA and LV-Luc/FH were produced at PackGene Biotech. Lentivirus transduction was performed as previously described (48). GL261 cells were transduced with LV-Luc/FH or LV- Luc/FH shRNA at a multiplicity of infection of 10. To enhance transduction efficiency, 2 μg/ml polybrene was added during the process. Transduced GL261 cells were then selected with 2.5 μg/ml puromycin for 1 week to ensure stable integration. The efficiency of lentivirus with overexpression or knockdown of FH was verified by Western blot analysis.

### GBM orthotopic models

For the orthotopic xenograft model, 8-week-old male C57BL/6J mice were intracranially injected with the transduced GL261 cells. In brief, the mice were anesthetized with 3% isoflurane in an induction chamber, and anesthesia was sustained by applying 2% isoflurane through a nose adaptor. 5 × 10^4^ cells in 3 μl PBS were injected into the right corpus striatum (coordinates: 1.5 mm anterior, 2 mm lateral to bregma, 2.5 mm depth from the dura) of the mice *via* a 701 N, 26s-gauge, 51mm, point style 10 μl Hamilton needle (with an injection duration of 15 min and a 3 min retention). The mice were housed at a density of five per cage and maintained under specific pathogen-free conditions in a 12/12 h light and dark cycle. Bioluminescent imaging was employed to monitor the intracranial GBM growth in mice on a weekly basis by intraperitoneal injection of D-luciferin potassium salt (150 mg/kg, Aladdin, L120798), followed by signal capture using the Spectrum IVIS imaging system (PerkinElmer). The mice were euthanized when either a 25% body weight loss or neurological symptoms (lethargy, ataxia, and seizures) were observed. The brains were harvested and subsequently frozen at −80 °C or fixed in 4% PFA, to be utilized for further analysis. All animal experiments were conducted in accordance with the institutional ethical guidelines for animal care, with the approval of the Animal Experimentation Ethics Committee of Nantong University (Approval ID: SYXK (SU) 2017-0046).

### Bioinformatics analysis

We performed comprehensive bioinformatics analyses using R packages to evaluate the prognostic and diagnostic value of target genes in glioma. Differential expression was analyzed using “*ggplot2*”. Survival outcomes were assessed *via* Kaplan-Meier analysis (“*survminer*”, “*survival*”) and Cox regression (“*survival*”; false discovery rate <0.05). Drug sensitivity (IC50) was predicted using “pRRophetic” (GDSC database). Time-dependent ROC analysis (“*timeROC*”, “*survivalROC*”) evaluated 1/3/5-years prognostic accuracy, while diagnostic ROC curves (“*pROC*”) determined optimal FH/TRIM17 cutoffs. A TRIM17-integrated nomogram was developed (“*rms*”) to predict 1-year survival. All models were validated through bootstrapping and calibration tests.

### Statistical analysis

All experiments were repeated at least three times independently. Data are presented as mean ± SD. Statistical significance between groups was determined using either ANOVA or paired/unpaired Student’s *t*-tests, as appropriate. For survival data, log-rank test was chosen and the result was displayed in the form of Kaplan-Meier plots. Besides, univariate and multivariate Cox regression analysis were used to assess the independent prognostic value of factors. Statistical analyses were performed using GraphPad Prism version 8.0 (https://www.graphpad.com/scientific-software/prism/) (GraphPad Software) or R software (https://www.r-project.org/) (version 3.6.2). A *p*-value < 0.05 was considered statistically significant.

## Data availability

The protein mass spectrometry raw data for TRIM17 succination have been deposited at ProteomeXchange with identifier PXD067085. The data analyzed in this study were obtained from the GBM, Low-Grade Glioma, and Glioma datasets in The Cancer Genome Atlas (http://portal.gdc.cancer.gov/), the Chinese Glioma Genome Atlas (CGGA; http://www.cgga.org.cn/), and the GEPIA database (http://gepia.cancer-pku.cn/). Values for all data points in graphs are reported in the Supporting Data Values file. Raw western blots are available in the Supplemental file.

## Supporting information

This article contains supporting information.

## Conflict of interest

The authors declare that they do not have any conflict of interest with the content of this article.
