# Supplementary material for: Fumarate hydratase suppresses glioblastoma progression by restricting fumarate-mediated succination of TRIM17
Source: J Biol Chem. 2026 Aug 7;302(9):113401. doi: 10.1016/j.jbc.2026.113401 (PMC13545428; doi:10.1016/j.jbc.2026.113401)
Supplement: Supplementary file 1 [file mmc1.docx]

**Supporting information**

**Fumarate hydratase suppresses glioblastoma progression by** **restricting fumarate-mediated succination of TRIM17**

Qianqian Ju, Yan Hu, Chiheng Gong, Runze Wu, Mujie Shen, Xuejian Wang, Xin Tang, Jianhong Shen, Cheng Sun

**This PDF file includes:**

Supplementary Figure S1 to S18

Supplementary Table S1 to S2

**Figure S1. FH negatively regulates GBM cell proliferation, migration and invasion.**

(A) FH overexpression in GBM cells. U118 and GL261 cells were transfected with a plasmid expressing *FH* for 24 h. *n =* 3 biologically independent replicates.

(B) Exogenous FH was expressed in mitochondria of U118 cells. Cells were transfected with a plasmid expressing FH-Flag. After 24 h, cells were subjected to immunofluorescence analysis using anti-Flag (red) and anti-TOM20 (green) antibodies. Hoechst (blue) was used to stain the nucleus. Scale bar = 20 µm.

(C) FH overexpression reduces U118 cell viability. *n =* 10 biologically independent replicates.

(D) FH overexpression decreases *Ki67* expression in U118 cells. *n =* 3 biologically independent replicates.

(E) FH overexpression reduces clonogenic growth in GL261 cells. *n =* 3 biologically independent replicates.

(F, G) FH overexpression inhibits cell migration and invasion in U118 (F) and GL261 (G) cells. Cell migration and invasion were analyzed by transwell assays. Scale bar = 50 µm. *n =* 3 biologically independent replicates.

(H, I) Wound-healing assays showing that FH overexpression attenuates cell migration in U118 (H) and GL261 (I) cells. Scale bar = 100 µm. *n =* 3 biologically independent replicates.

Protein levels were analyzed by western blot using Actin served as a loading control. EV: empty vector. Data are presented as mean ± SD. Statistical significance was tested by unpaired Student’s *t*-test. **P* < 0.05, ***P* < 0.01 and ****P* < 0.001.

**Figure S2. Catalytically inactive FH mutants fail to inhibit GBM cell viability, migration, and invasion.**

(A) FH D238H and FH E378K fail to suppress cell viability in U251 cells. Cells were transfected with plasmids expressing wild-type FH, FH D238H, or FH E378K for 24 h. *n =* 10 biologically independent replicates.

(B-D) Transwell assays showing that FH D238H and FH E378K mutants loss inhibitory effects on cell migration and invasion in U251 cells. Scale bar = 50 µm. *n =* 3 biologically independent replicates.

(E, F) Wound-healing assays showing that FH D238H and FH E378K mutants fail to inhibit cell migration in U251 cells. Scale bar = 100 µm. *n =* 3 biologically independent replicates.

(G) Overexpression of wild-type FH, FH D238H, and FH E378K in U251 cells. Protein levels were analyzed by western blot using Actin as a loading control. *n =* 3 biologically independent replicates.

EV: empty vector. WT: wild-type. Data are presented as mean ± SD. Statistical significance was analyzed by one-way ANOVA with Dunnett’s multiple comparisons test. ns: no significance, ***P* < 0.01, and ****P* < 0.001.

**Figure S3.** **FH silencing promotes cell growth and metastasis in U87 cells.**

(A, B) FH knockdown in GBM cells. U87 cells were transfected with *FH* siRNAs. Forty-eight hours post-transfection, FH expression was measured by qRT-PCR (A, *n =* 4 biologically independent replicates) or western blot (B, *n =* 3 biologically independent replicates).

(C) FH knockdown enhances GBM cell viability. Cell viability was assessed at 72 h post-transfection. *n* = 10 biologically independent replicates.

(D) *Ki67* expression is elevated upon FH knockdown in GBM cells. *n* = 3 biologically independent replicates.

(E) FH knockdown stimulates clonogenicity. *n* = 3 biologically independent replicates.

(F) Transwell assays show enhanced migration and invasion following FH knockdown. Scale bar = 50 µm. *n* = 3 biologically independent replicates.

(G) Wound-healing assays showing that FH knockdown promotes GBM cell migration. Scale bar = 100 µm. *n* = 3 biologically independent replicates.

Protein levels were analyzed by western blot using Actin as a loading control. Gene expression was analyzed by qRT-PCR with *18S rRNA* as the internal control. NC: negative control. Data are presented as mean ± SD. Statistical significance was determined using an unpaired Student’s *t*-test. * *P* < 0.05, ** *P* < 0.01, and *** *P* < 0.001 versus NC.

**Figure S4. FH knockdown promotes cell viability, migration, and invasion in U251 cells.**

(A, B) Validation of FH knockdown in U251 cells. Cells were transfected with *FH* siRNAs for 48 h and subsequently harvested for qRT-PCR (A; *n =* 4 biologically independent replicates) or western blot analysis (B; *n = 3* biologically independent replicates).

(C) FH knockdown increases cell viability. Cell viability was assessed at 72 h after transfection. *n* = 10 biologically independent replicates.

(D) FH knockdown upregulates *Ki67* expression. *n* = 3 biologically independent replicates.

(E) FH knockdown enhances clonogenic growth. *n* = 3 biologically independent replicates.

(F) Transwell assays showing that FH knockdown promotes cell migration and invasion. Scale bar = 50 µm. *n* = 3 biologically independent replicates.

(G) Wound-healing assays showing that FH knockdown enhances cell migration. Scale bar = 100 µm. *n* = 3 biologically independent replicates.

Protein levels were analyzed by western blot using Actin as a loading control. Gene expression was analyzed by qRT-PCR with *18S rRNA* as the internal control. NC: negative control. Data are presented as mean ± SD. Statistical significance was determined using an unpaired Student’s *t*-test. * *P* < 0.05, ** *P* < 0.01, and *** *P* < 0.001 versus NC.

**Figure S5. FH inhibition promotes cell proliferation of GBM cells.**

U87, U251, and GL261 cells were treated with fumarate hydratase-IN-1 (FHi; 10 µM) for 24 h, followed by assessment of cell viability and clonogenic growth.

(A) FH inhibition increases the cell viability of GBM cells. *n =* 10 biologically independent replicates.

(B) FH inhibition enhances clonogenic growth of GBM cells. *n =* 3 biologically independent replicates.

Data are presented as mean ± SD. Statistical significance was determined using an unpaired Student’s *t*-test. **P* < 0.05, ***P* < 0.01 and ****P* < 0.001.

**Figure S6. FH inhibition promotes migration and invasion of GBM cells.**

U87, U251, and GL261 cells were treated with fumarate hydratase-IN-1 (FHi; 10 µM) for 24 h, followed by assessment of cell migration and invasion.

(A) Transwell assays showing that FH inhibition promotes the migration and invasion of GBM cells. Scale bar = 50 µm. *n =* 3 biologically independent replicates.

(B) Wound-healing assays showing that FH inhibition increases cell migration. Scale bar = 100 µm. *n =* 3 biologically independent replicates.

Data are presented as mean ± SD. Statistical significance was determined using an unpaired Student’s *t*-test. **P* < 0.05, ***P* < 0.01 and ****P* < 0.001.

**Figure S7. FH overexpression abolishes the promotive effects of FH knockdown on cell viability, migration, and invasion in U251 cells.**

(A) The increase in cell viability induced by FH knockdown was reversed by FH overexpression. U251 cells were transfected with *FH* siRNA-3# for 24 h and subsequently transfected with a plasmid expressing siRNA-resistant FH for an additional 24 h. *n* = 10 biologically independent replicates.

(B-D) Transwell assays showing that siRNA-resistant FH overexpression abolishes the FH knockdown-induced increases in cell migration and invasion. Scale bar = 50 µm. *n* = 3 biologically independent replicates.

(E, F) Wound-healing assays showing that siRNA-resistant FH overexpression counteracts the FH knockdown-induced enhancement of cell migration. Scale bar = 100 µm. *n* = 3 biologically independent replicates.

(G) siRNA-resistant FH overexpression counteracts FH knockdown. Protein levels were analyzed by western blot using Actin as a loading control. *n* = 3 biologically independent replicates.

Data are presented as mean ± SD. Statistical significance was determined using one-way ANOVA with Dunnett’s multiple comparisons test. ns: no significance, * *P* < 0.05, ** *P* < 0.01, and *** *P* < 0.001.

**Figure S8. FH regulates intracellular fumarate levels in GBM cells.**

(A) FH overexpression decreases intracellular fumarate levels in GBM cells 24 h post-transfection. *n = 5* biologically independent replicates.

(B) FH knockdown increases intracellular fumarate levels in U87 and U251 cells 72 h post-transfection. *n = 5* biologically independent replicates.

(C) Pharmacological inhibition of FH increases fumarate levels in GBM cells. U87 and U251 cells were treated with FH inhibitor (FHi; 10 µM) for 24 h. *n = 3* biologically independent replicates.

EV: empty vector. Data are presented as mean ± SD. Statistical significance was determined by unpaired Student’s *t*-test (A, B) or one-way ANOVA with Dunnett’s multiple comparisons test (C). * *P* < 0.05, ** *P* < 0.01 and *** *P* < 0.001.

**Figure S9. DMF induces TRIM17 succination and promotes its degradation.**

(A) Quantification of 2SC levels shown in Fig. 3E.

(B) Quantification of 2SC levels shown in Fig. 3F.

(C) Quantification of 2SC levels shown in Fig. 3G.

(D) Catalytically inactive FH mutants have no effect on global protein succination in U251 cells. Cells were transfected with plasmids expressing FH D238H or FH E378K for 24 h.

(E) TRIM17 promotes MCL1 ubiquitination in U251 cells. U251 cells were transfected with a TRIM17-expressing plasmid. Twelve hours after transfection, cells were treated with DMF (10 μM) for an additional 12 h. Following treatment, cell lysates were subjected to immunoprecipitation using an anti-MCL1 antibody. The resulting immunocomplexes were analyzed by immunoblotting with antibodies against ubiquitin, TRIM17, and MCL1.

IP: immunoprecipitation. IB: immunoblot. Protein levels were analyzed by western blot using Actin as a loading control. Data are presented as mean ± SD. Statistical significance was determined by unpaired Student’s *t*-test. ns: no significance; * *P* < 0.05, ** *P* < 0.01 and *** *P* < 0.001 versus HA1800, C8D1A, EV, or DMSO.

**Figure S10. TRIM17 succination.**

(A) Identification of succinated proteins by LC-MS/MS. Protein extracts from DMF-treated U87 cells were subjected to LC-MS/MS analysis to detect proteins with succination modifications (cysteine residues with +116 Da mass shift).

(B) FH overexpression reduces TRIM17 succination in U251 cells. Cells were transfected with an empty vector (EV) or a plasmid expressing FH for 24 h. Cells lysates were then subjected to immunoprecipitation (IP) using an anti-TRIM17 antibody. The resulting immunocomplexes, together with total cell lysates (Input), were analyzed by immunoblotting (IB) with anti-2SC, anti-TRIM17, and anti-Actin antibodies.

(C) DMF treatment induces TRIM17 succination in U251 cells. Cells were treated with DMF (10 µM) for 24 h and subsequently subjected to immunoprecipitation (IP) using an anti-TRIM17 antibody. The resulting immunocomplexes, together with total cell lysates (Input), were analyzed by immunoblotting using anti-2SC, anti-TRIM17, and anti-Actin antibodies.

**Figure S11. TRIM17 C370S counteracts the effects of FH knockdown on cell viability, migration, and invasion in U251 cells.**

(A) TRIM17 C370S overexpression abolishes the FH knockdown-induced increase in cell viability. U251 cells were transfected with *FH* siRNA-3# for 24 h and subsequently transfected with a plasmid expressing FH C370S for an additional 24 h. *n =* 10 biologically independent replicates.

(B-D) Transwell assays showing that TRIM17 C370S overexpression counteracts the FH knockdown-induced increases in cell migration and invasion. Scale bar = 50 µm. *n =* 3 biologically independent replicates.

(E, F) Wound-healing assays showing that TRIM17 C370S overexpression reverses the FH knockdown-induced enhancement of cell migration. Scale bar = 100 µm. *n =* 3 biologically independent replicates.

(G) FH and TRIM17 protein levels, as analyzed by western blot using Actin as a loading control. *n* = 3 biologically independent replicates.

Data are presented as mean ± SD. Statistical significance was determined by one-way ANOVA with Dunnett’s multiple comparisons test. * *P* < 0.05, ** *P* < 0.01 and *** *P* < 0.001.

**Figure S12. TRIM17 knockdown reduces global protein ubiquitination in U251 cells.**

(A) Validation of TRIM17 knockdown in U251 cells. Cells were transfected with *Trim17* siRNAs for 48 h, and subsequently harvested for western blot analysis. *n =* 3 biologically independent replicates.

(B) TRIM17 knockdown decreases global protein ubiquitination. U251 cells were transfected with *Trim17* siRNA-3# for 48 h and subsequently harvested for western blot analysis.

NC: negative control. Protein levels were analyzed by western blot using Actin as a loading control. Data are presented as mean ± SD. Statistical significance was determined by one-way ANOVA with Dunnett’s multiple comparisons test. * *P* < 0.05 and *** *P* < 0.001 versus NC.

**Figure S13. Fumarate derivatives MMF and DMF reduce TRIM17 protein stability, while FH increases its stability in U251 cells.**

(A and B) MMF and DMF decrease TRIM17 protein stability. U251 cells were transfected with a plasmid expressing *TRIM17.* After 24 h, MMF (250 µM) or DMF (10 µM) was added in culture medium for either 24 h or 4 h. Then the cells were treated with cycloheximide (CHX, 50 μg/ml) and harvested at different time points as indicated. *n =* 3 biologically independent replicates.

(C) FH overexpression extends TRIM17 protein stability. U251 cells were transfected with a plasmid expressing *TRIM17*, together with empty vector (EV) and *FH* as indicated*.* After 24 h, cells were treated with cycloheximide (CHX, 50 μg/ml) with different exposure time as indicated. *n = 3* biologically independent replicates.

(D) DMF-induced TRIM17 degradation occurs via the proteasome and lysosome pathways. U251 cells were treated with DMF (10 µM), MG132 (10 µM), or chloroquine (CQ; 10 µM) as indicated for 24 h and subsequently harvested and subjected to western blot analysis.

Protein levels were analyzed by western blot using Actin as a loading control. Data are presented as mean ± SD. Statistical significance was determined using two-way ANOVA with Šidák’s post-hoc test (A-C) or one-way ANOVA with Dunnett’s multiple comparisons test (D). **P* < 0.05, ***P* < 0.01 and ****P <* 0.001.

**Figure S14. TRIM17 knockdown promotes cell viability, migration, and invasion in U251 cells.**

(A) TRIM17 knockdown increases cell viability in U251 cells. Cells were transfected with *Trim17* siRNA-3# for 48 h. *n =* 10 biologically independent replicates.

(B, C) TRIM17 knockdown increases cell migration and invasion, as assessed by Transwell assays. Scale bar = 50 µm. *n =* 3 biologically independent replicates.

(D, E) Wound-healing assays showing that TRIM17 knockdown promotes cell migration in U251 cells. Scale bar = 100 µm. *n =* 3 biologically independent replicates.

Data are presented as mean ± SD. Statistical significance was determined by unpaired Student’s *t*-test. ** *P* < 0.01 and *** *P* < 0.001 versus NC.

**Figure S15. TRIM17 knockdown counteracts the FH-induced suppression of cell viability, migration, and invasion in U251 cells.**

(A) TRIM17 knockdown abolishes the FH-induced reduction in cell viability. U251 cells were transfected with *Trim17* siRNA-3# for 24 h and subsequently transfected with a plasmid expressing FH for an additional 24 h. *n =* 10 biologically independent replicates.

(B-D) TRIM17 knockdown reverses FH-induced suppression of cell migration and invasion, as assessed by Transwell assays. Scale bar = 50 µm. *n =* 3 biologically independent replicates.

(E, F) Wound-healing assays showing that TRIM17 knockdown abolishes the FH-induced decrease in cell migration. Scale bar = 100 µm. *n =* 3 biologically independent replicates.

(G) Protein levels of FH and TRIM17. Protein levels were analyzed by western blot using Actin as a loading control. *n* = 3 biologically independent replicates.

Data are presented as mean ± SD. Statistical significance was determined by one-way ANOVA with Dunnett’s multiple comparisons test. * *P* < 0.05, ** *P* < 0.01, and *** *P* < 0.001.

**Figure S16.** **TRIM17 is downregulated in glioma and associated with poor prognosis, chemotherapy sensitivity, and diagnostic value.**

(A) CGGA database analysis indicates a progressive decline in TRIM17 expression with increasing glioma grade.

(B-D) Kaplan-Meier survival analyses show that low TRIM17 expression correlates with shorter overall survival (OS) in LGG and glioma patients (B, TCGA; C, GEPIA; D, CGGA).

Data are presented as mean ± SD. One-way ANOVA followed by Dunnett's multiple comparisons test (A), or log-rank test (B-D).

**Figure S17. Overexpression of FH suppresses GBM progression in mice.**

(A) FH protein levels were increased by lentiviral overexpression (LV-FH). GL261 cells were transduced with LV-Luc/CON or LV-Luc/FH for 48 h. *n* = 3 biologically independent replicates.

(B) Representative *in vivo* bioluminescent images of C57BL/6J mice bearing GL261 orthotopic xenografts. GL261 cells were transduced with LV-Luc/CON or LV-Luc/FH. After 48 h, transduced cells were selected using puromycin (2.5 µg/ml), and then injected intracranially into C57BL/6J mice. *n* = 10 biologically independent animals.

(C) Body weight of mice was not altered between LV-CON and LV-FH groups.

Protein expression was assessed by western blot using Actin as a loading control. Data are presented as mean ± SD. Statistical significance was tested by unpaired Student’s *t*-test (A) and two-way ANOVA followed by Šidák’s post-hoc test (C). ****P* < 0.001.

**Figure S18. FH knockdown promotes GBM progression in mice.**

(A) FH protein levels were reduced by lentiviral knockdown. GL261 cells were transduced with LV-Luc/shNC or LV-Luc/shFH for 48 h. *n* = 3 biologically independent replicates.

(B) Representative *in vivo* bioluminescent images of C57BL/6J mice bearing GL261 orthotopic xenografts. GL261 cells were transduced with LV-Luc/shNC or LV-Luc/shFH. After 48 h, transduced cells were selected using puromycin (2.5 µg/ml), and then injected intracranially into C57BL/6J mice. *n* = 10 biologically independent animals.

(C) Body weight was not affected by FH knockdown.

Protein expression was assessed by western blot using Actin as a loading control. Data are presented as mean ± SD. Statistical significance was tested by unpaired Student’s *t*-test (A) and two-way ANOVA followed by Šidák’s post-hoc test (C). ****P* < 0.001.

**Table S1.** The sequences used in gene knockdown and mutation.

| **Genes** | **Sequences (5’-3’)** |
| --- | --- |
| NC siRNA | UUCUCCGAACGUGUCACGU |
| *FH* siRNA-1 | UUAAAGUUCAUCGUAGAUCTT |
| *FH* siRNA-2 | UUUAUUAACAUGAUCGUUGTT |
| *FH* siRNA-3 | UUUAUUAACAUGAUCGUUGGGTT |
| *FH* shRNA | CCCAAUGAUCAUGUUAACAAA |
| *TRIM17* siRNA-1 | UAGCACUUCAAUCUGUCCGTT |
| *TRIM17* siRNA-2 | UCAAACUCCAGCACAAUGCTT |
| *TRIM17* siRNA-3 | UCUUCUUCCGUCUCCAGAGTT |
| *TRIM17* (C370S) | F: TTGTGGGCCCTGGGTGTGAGCAGGGACAACGTGAGCCG |
|  | R: GCAACACCCGGGACCCACACACGTCCCTGTTGCACT |
| *FH* D238H | F: TACTCATACTCAGCATGCTGTTCCACTTA |
|  | R: AAGAGTAAGTGGAACAGCATGCTGAGTAT |
| *FH* E378K | F: TGAACCCTACTCAGTGTAAAGCAATGA |
|  | R: AACCATGGTCATTGCTTTACACTGAGTA |
| siRNA-resistant FH | F: TGCATCCGAATGACCACGTGAACAAGAGCCAGAGCTC |
|  | R: CTTGTTCACGTGGTCATTCGG ATGCACAGGTATCTTGCTG |

F: forward; R: reverse.

**Table S2.** Primer sequences used in qRT-PCR.

| **Genes** | **Primer sequences (5’-3’)** |
| --- | --- |
| *FH*-human | F: CAGTTTGACGAATGGGTAAA |
|  | R: ATCTTAGACCTAGCACATCC |
| *TRIM17*-human | F: CACCTTCTCTGCCCTAAC |
|  | R: CATGGTCACTGTGGAGAT |
| *Ki67-*human | F: CACTAACTCTGTACCTACCAT |
|  | R: GAACAATTCCAGGCAAACTA |
| *18S rRNA* | F: AGCTCCAATAGCGTATATTAAAG |
|  | R: CGGTCCTATTCCATTATTCCTA |

F: forward; R: reverse.
